# Efficacy of Quinidine in Brugada Syndrome: A Literature Review

**DOI:** 10.31083/RCM51365

**Published:** 2026-10-12

**Authors:** George Bazoukis, Emmanouil Andreakis, Athanasios Saplaouras, Tong Liu, Gary Tse, Konstantinos P. Letsas, Michael Efremidis, Adrian Baranchuk

**Affiliations:** ^1^Department of Basic and Clinical Sciences, University of Nicosia Medical School, 2417 Nicosia, Cyprus; ^2^Department of Cardiology, Larnaca General Hospital, State Health Services Organization, 6301 Larnaca, Cyprus; ^3^Arrhythmia Unit, Onassis Cardiac Surgery Center, 17674 Athens, Greece; ^4^Tianjin Key Laboratory of Ionic-Molecular Function of Cardiovascular Disease, Department of Cardiology, Tianjin Institute of Cardiology, Second Hospital of Tianjin Medical University, 300150 Tianjin, China; ^5^Kent and Medway Medical School, University of Kent and Canterbury Christ Church University, 927700 Canterbury, Kent, UK; ^6^School of Nursing and Health Studies, Hong Kong Metropolitan University, Hong Kong, China; ^7^Division of Cardiology, Kingston Health Science Center, Queen’s University, Kingston, ON K7L 3N6, Canada

**Keywords:** quinidine, Brugada syndrome, sudden cardiac death, ventricular arrhythmias

## Abstract

Brugada syndrome (BrS) is an inherited arrhythmogenic disorder associated with an increased risk of ventricular fibrillation (VF) and sudden cardiac death. Quinidine and hydroquinidine are currently used to prevent ventricular arrhythmias in BrS. This review summarizes the available evidence on the efficacy, safety, and clinical applications of these drugs in BrS. Ten studies involving more than 300 patients with BrS were analyzed. Quinidine or hydroquinidine therapy prevented VF inducibility in 42.9–90% of cases and reduced recurrent arrhythmic events by approximately 60–70%. Among patients with implantable cardioverter-defibrillators (ICDs), reductions in both appropriate and inappropriate shock counts were observed. Gastrointestinal intolerance was the most common side effect, occurring in 20–30% of patients, with drug discontinuation in up to 26%. QT prolongation was infrequent, and serious proarrhythmic events were rare. Despite these benefits, quinidine remains unavailable or difficult to access in many regions, limiting its widespread clinical use. In conclusion, quinidine and hydroquinidine exert antiarrhythmic effects in BrS and reduce the recurrence of ventricular arrhythmias, particularly among ICD-treated or high-risk patients. Although therapy is constrained by gastrointestinal intolerance and limited availability, low-dose regimens may be better tolerated while maintaining efficacy. These agents can be considered, in selected patients, as adjuncts or alternatives to device therapy; meanwhile, further multicenter studies are warranted to confirm long-term safety, determine optimal dosing, and improve global accessibility.

## 1. Introduction

Brugada syndrome (BrS) is a heritable cardiac channelopathy that is associated with an increased risk of sudden cardiac death (SCD) due to malignant ventricular arrhythmias [1]. Although implantable cardioverter-defibrillator (ICD) use is the cornerstone of management for patients with an aborted SCD or spontaneous sustained ventricular tachycardia, the management of asymptomatic patients remains challenging [2,3]. Several variables have been proposed for risk stratification in asymptomatic patients, including a spontaneous type 1 electrocardiographic (ECG) pattern, first-degree atrioventricular (AV) block, QRS fragmentation, an S wave in leads I and II and prolonged QRS duration [4,5]. Pharmacological management can play an additional role in preventing ventricular arrhythmias in BrS. Quinidine has been shown to reduce recurrence of life-threatening arrhythmias in patients with BrS with aborted cardiac arrest [6,7]. However, quinidine use requires careful monitoring due to potential adverse effects, including gastrointestinal disturbances and proarrhythmia [6,7]. Quinidine has a long history in BrS management, beginning with early case reports and small series in the 1990s that suggested Class IA agents could suppress ventricular arrhythmias in this condition [8]. Over the 2000s, observational cohorts further documented quinidine’s ability to reduce inducible ventricular arrhythmias and ICD therapies [9]. This study aims to explore and summarize the existing data on the efficacy of quinidine in managing patients with BrS.

## 2. Methods/Search Strategy

This narrative review used a structured literature search to identify clinical evidence evaluating quinidine or hydroquinidine in the management of BrS. PubMed and Google Scholar were searched using combinations of the following keywords: “*Brugada syndrome*”, “*quinidine*”, “*hydroquinidine*”, “*ventricular arrhythmia*”, and “*ICD shocks*”*.* Reference lists of relevant publications, review articles, and guideline documents were also screened to identify additional studies. Eligible studies included randomized controlled trials, observational cohorts, registry analyses, electrophysiological studies, and case series reporting clinical or electrophysiological outcomes following quinidine-based therapy. Studies published in languages other than English were excluded. Because of heterogeneity in study design, population, endpoints, and follow-up duration, the evidence was synthesized narratively rather than through formal meta-analysis.

## 3. Evidence From Randomized Controlled Trials (RCT), Observational Studies and Case Series

The baseline characteristics of the included studies are summarized in Table 1, Ref. [6,10,11,12,13,14,15,16,17,18].

**Table 1.** **Summary of studies evaluating the efficacy and tolerability of quinidine or hydroquinidine therapy in patients with Brugada syndrome**.

First author (year)	Study design/population	Therapy	Main outcomes	Adverse effects/follow-up
Hermida (2004) [14]	Observational; 35 participants (31 asymptomatic, 4 with ICDs)	Hydroquinidine 600 mg/day	VF inducibility suppressed in 76%; no ICD recurrences	GI intolerance (8%); follow-up 14 months
Bouzeman (2014) [11]	Prospective cohort; 44 asymptomatic inducible individuals	Hydroquinidine 600 mg/day	Non-inducibility achieved in 77%; annual arrhythmic event rate 1.04%	Discontinuation 18% (due to GI intolerance/QT prolong.); 6.6 years
Belhassen (2015) [12]	Registry; 96 individuals (10 SCD survivors, 27 syncope, 59 asymptomatic)	Quinidine or HQ 200–600 mg twice daily	90% became non-inducible; no arrhythmic events reported during therapy	GI/cinchonism 38%, ~20% stopped; up to 33 years
Anguera (2016) [15]	Nationwide survey; 29 with recurrent ICD shocks	Quinidine ~590 mg or HQ ~700 mg/day	ICD shocks reduced from 6 to 0 (*p* < 0.0001); 66% shock-free	QT prolong. (2 cases); 60 months
Andorin (2017, QUIDAM) [10]	RCT; 50 with ICDs	Hydroquinidine 738 mg/day vs placebo	No events on HQ; 2 events on placebo	Discontinuation 26% (GI/QT); 6 months + ext.
Shen (2017) [16]	Case series; 10 with ICDs	Quinidine ≤200 mg/day	Nine of ten patients remained free of ventricular arrhythmias on therapy	Mild leukopenia (1 case); 22 months
Kaneko (2014) [17]	Retrospective; 22 presenting with electrical storm	Quinidine or bepridil (adjunctive)	VF storms suppressed; quinidine prevented further recurrence	Occasional GI intolerance; 6 years
Mazzanti (2019) [6]	Observational cohort; 53 treated vs 441 controls (secondary prevention)	Quinidine 439 mg/day	Arrhythmic events reduced 14.7%→3.9% (*p* = 0.03)	GI intolerance occurred ~25%; 5 years
Mizusawa (2006) [13]	Electrophysiologic; 14 participants	Quinidine 300–600 mg/day	VF inducibility suppressed 42.9%; no recurrence in continuers	Diarrhea 12.5%; 31 months
Márquez (2012) [18]	Case series; 6 patients with ICDs	≤600 mg/day of quinidine sulfate or hydroquinidine	No arrhythmia recurrence	No side effects; Median follow-up 4 years (from 2 to 8 years)

Abbreviations: VF, ventricular fibrillation; VA, ventricular arrhythmia; ICD, implantable cardioverter-defibrillator; HQ, hydroquinidine; GI, gastrointestinal; QT, QT interval prolongation; SCD, sudden cardiac death; RCT, Randomized Controlled Trials.

The QUIDAM study was a prospective, randomized, double-blind, multicentre trial that examined the role of hydroquinidine in preventing life-threatening ventricular arrhythmias in high-risk patients with BrS [10]. The study included 50 patients with an ICD for one of the following indications: prior aborted SCD or documented ventricular tachyarrhythmias, unexplained syncope of an arrhythmic origin, or spontaneous type 1 BrS ECG pattern with a positive EPS [10]. The mean dose of hydroquinidine was 738 mg/day. Thirty-four patients experienced hydroquinidine-related side effects, mainly gastrointestinal, leading to discontinuation of the therapy in 13 patients. During follow-up, no arrhythmic events occurred amongst patients receiving hydroquinidine. By contrast, one appropriate ICD shock and one self-terminating episode of ventricular fibrillation (VF) occurred under placebo therapy [10]. Although these data suggest hydroquinidine may prevent future ventricular arrhythmias, a statistically significant difference between the two groups was not observed, mainly because of the very low incidence of arrhythmic events in the enrolled population. Moreover, 26% of patients discontinued therapy due to hydroquinidine-related side effects [10].

Mazzanti et al. [6] compared the clinical outcomes of 53 patients treated with quinidine to untreated controls with BrS. Of the 53 patients, 27 patients received quinidine for secondary prevention after one or more episodes of life-threatening ventricular arrhythmias. Hydroquinidine was administered at a median dose of 450 mg/day. The study showed that patients who received quinidine had a non-significant 26% reduction in the risk of a first life-threatening arrhythmic event during follow-up (HR = 0.74; 95% CI, 0.22–2.48; *p* = 0.62) [6]. In 123 patients with a previous history of life-threatening arrhythmias, the annual rate of recurrent life-threatening arrhythmias was reduced from 14.7% off-quinidine to 3.9% on-quinidine (HR = 0.33; 95% CI, 0.12–0.92; *p* = 0.03). However, recurrences of life-threatening arrhythmias still occurred in 4 (14.8%) symptomatic patients while on treatment [6]. Thus, altogether low-dose quinidine was generally well tolerated and reduced the occurrence of fatal arrhythmias in patients treated for secondary prevention. However, approximately 15% of these patients experienced a recurrent fatal arrhythmic event while on treatment, so quinidine cannot replace the ICD [6].

Bouzeman et al. [11] studied 44 asymptomatic BrS patients with inducible VF during the EPS. All patients received hydroquinidine and underwent a second EPS while on hydroquinidine. Ten patients remained inducible on hydroquinidine whereas 34 patients were not inducible. In this group, an ICD was ultimately implanted in four patients (12%), due to the occurrence of syncope in two and to major hydroquinidine intolerance in two others, with the occurrence of appropriate ICD therapy in one out of the four patients. Of the 10 patients on hydroquinidine who still had inducible arrhythmias, 9 received an ICD while no appropriate ICD therapies occurred during follow-up. This study demonstrates a very low annual incidence of arrhythmic events under hydroquinidine in asymptomatic BrS patients with positive EPS. Furthermore, residual inducibility on hydroquinidine did not predict future arrhythmic events.

Belhassen et al. [12] examined the 96 patients with BrS (59 asymptomatic, 10 cardiac arrest survivors and 27 patients with syncope) who underwent EPS. Sixty-six patients were inducible while 60 of them had a repeat EPS under quinidine (n = 54), disopyramide (n = 2), or both (n = 4). Forty-four of the 60 patients were noninducible on antiarrhythmic therapy, and 52 of the 58 patients who received quinidine were noninducible. No arrhythmic events occurred during follow-up in patients who received antiarrhythmic medication. Two patients who interrupted quinidine had a VF episode, but no arrhythmia recurrence was noted following the re-initiation of quinidine [12].

Mizusawa et al. [13] examined the role of low-dose quinidine on ventricular arrhythmias during EPS stimulation and during a subsequent 31-month follow-up period. Specifically, 14 BrS patients (9 asymptomatic patients) underwent EPS stimulation and VF was induced in all patients. Following oral quinidine administration (300 mg or 600 mg/day), ventricular fibrillation induction was prevented in 6 of 14 patients [13]. Over 31 months of follow-up, 9 patients (3 patients with a history of ventricular arrhythmia) received quinidine and no arrhythmia recurrence was observed [13].

Hermida et al. [14] studied 35 BrS patients who received hydroquinidine. Of these, four patients were prescribed this medication because of multiple appropriate ICD shocks and thirty-one asymptomatic patients were prescribed it due to the presence of inducible arrhythmias during EPS. In the latter group, a second EPS was performed under hydroquinidine in 29 patients (27 patients received 600 mg/day and 2 patients 900 mg/day) and 22 (76%) were non-inducible [14]. During follow-up, 1 of the 10 asymptomatic patients with ICD had an appropriate ICD shock; of the 21 asymptomatic patients without ICD, two had a syncope episode (one patient had unexplained syncope and one patient had syncope associated with a long QTc interval) while none of the four symptomatic patients had an arrhythmic event [14].

In a Spanish multi-centre study, 29 patients who received quinidine or hydroquinidine were analyzed. Specifically, 9 patients had electrical storm and 20 patients had frequent ICD shocks [15]. In this cohort, 66% of the patients remained free of appropriate ICD discharges after a mean follow-up period of 60 months on quinidine [15]. Other important findings were a significant reduction in the burden of ICD shocks with quinidine and that temporary discontinuation of quinidine was a significant predictor of recurrent shocks [15]. The effectiveness of quinidine has also been examined in a Chinese case series that included 10 BrS patients with a history of appropriate ICD shocks treated with quinidine (≤200 mg/day) [16]. Quinidine prevented recurrence of ventricular arrhythmias in nine patients while the patient with arrhythmia recurrence had discontinued quinidine due to anxiety. The re-initiation of quinidine prevented arrhythmia recurrence in this patient [16]. Kaneko et al. [17], examined 22 high-risk BrS patients who survived electrical storm. Over a mean follow-up of 6.4 years, 12 patients experienced VF recurrences after the first electrical storm [17]. Notably, 6 patients treated with bepridil, 100–200 mg daily, 2 patients treated with quinidine, 300–600 mg daily, and 1 patient treated with amiodarone, 100 mg daily, remained free from VF recurrences [17]. Márquez et al. [18] evaluated low-dose quinidine (≤600 mg/day) for preventing recurrent ventricular arrhythmias in BrS patients with ICDs. Six men from two referral centers who received quinidine (or hydroquinidine) after arrhythmic syncope or appropriate ICD shocks (four with arrhythmic storm) were followed for a median of 4 years (from 2–8 years); none had recurrent ventricular arrhythmias or reported side effects [18].

## 4. Clinical Perspective

Quinidine is a Class IA antiarrhythmic drug according to the Vaughan Williams classification. Quinidine’s antiarrhythmic effects are not limited to inhibition of sodium channels. During phase 0 of the cardiac action potential, quinidine—acting as a Class IA antiarrhythmic—binds within the open state of the rapid sodium channel (*I*_Na_) and leads to sustained blockade contributing to a reduction in conduction velocity in both the atrial and ventricular myocardium [19], whereas specialized conduction tissues may be less affected. During phase 1, quinidine also inhibits the transient outward current (*I*_to_) responsible for the notch in the early repolarization phase of the action potential which plays a significant role in arrhythmogenesis of BrS [20].

Existing data suggest a potential benefit of quinidine in preventing or reducing ventricular arrhythmic events in patients with BrS (Fig. 1). Lower-dose regimens may be better tolerated in selected patients. According to current guidelines, quinidine may be considered in patients with BrS who qualify for an ICD but have a contraindication, decline ICD implantation, or experience recurrent ICD shocks [21]. Although quinidine may provide clinically important benefit in the setting of BrS, it is not readily accessible in many countries. A survey published in 2013 reported that quinidine was readily available in only 19 countries (14%), not accessible in 99 countries (76%), and obtainable only through special regulatory processes in 13 countries (10%) requiring 4–90 days for approval [22].

**Fig. 1.**
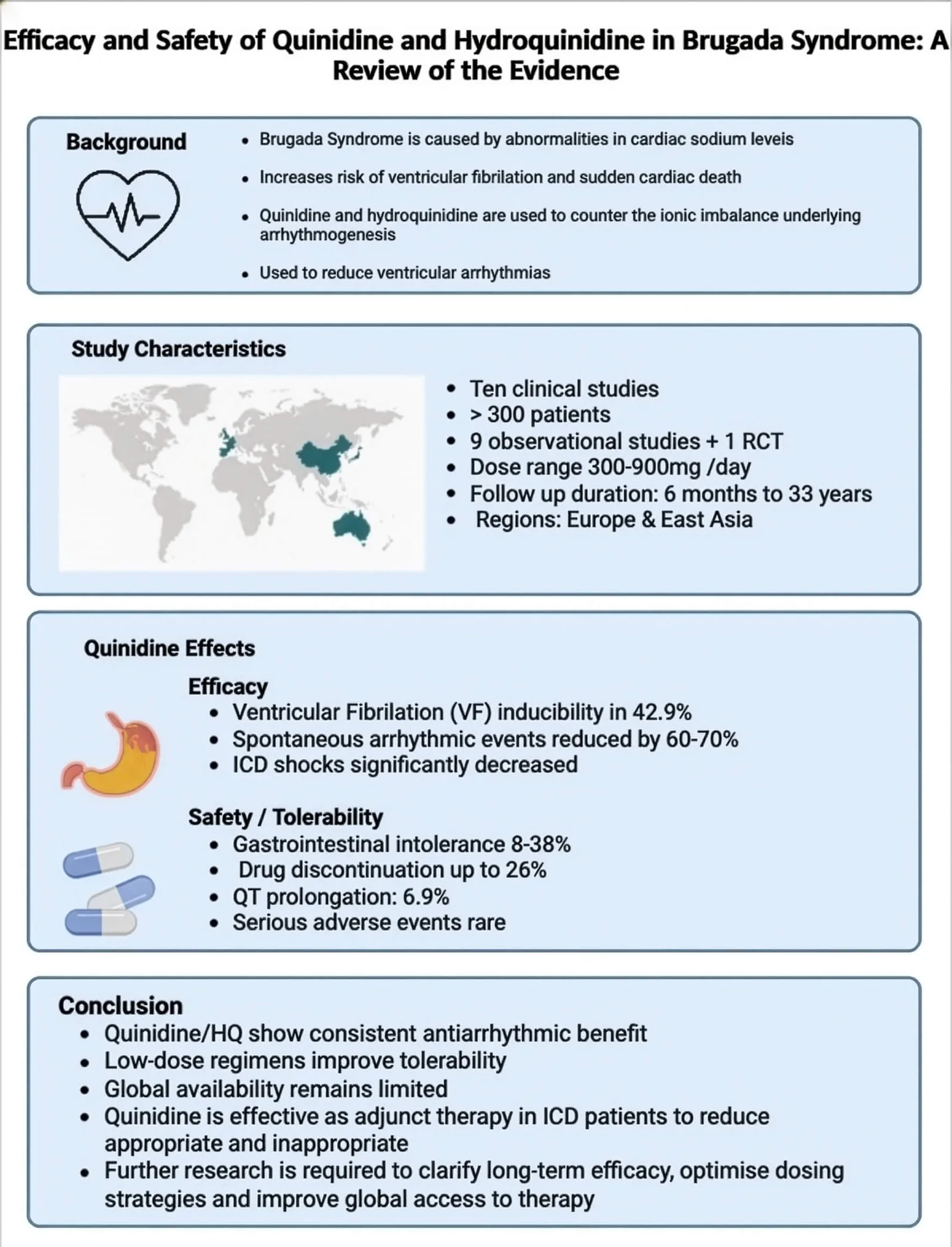
**Summary of the available evidence and clinical positioning of quinidine and hydroquinidine in Brugada syndrome**. Created in BioRender.

Gastrointestinal adverse events are the most frequent complications from quinidine use. Less common reactions include headache, tinnitus, photophobia, fever, hepatitis, leukopenia, thrombocytopenia, lupus-like syndrome, and QTc prolongation. The adverse effects of quinidine are dose-dependent; thus, patient tolerance can be improved by reducing the administered dose. Low-dose quinidine may retain efficacy while improving tolerability in selected patients [6,16]. In addition, administration at 21:00, according to the circadian distribution of VT/VF episodes, has been proposed as a strategy to optimize protection [17].

Beyond patient experience, clinical data further suggest the therapeutic role for quinidine in selected patients with BrS. Observational studies and one randomised controlled trial have reported suppression or reduction of ventricular arrhythmic events in selected BrS populations treated with quinidine or hydroquinidine. Early work by Hermida et al. [14] and Bouzeman et al. [11] showed prevention of VF inducibility in 76–77% of cases, while long-term follow-up data [9,12] suggested sustained arrhythmic benefit during extended follow-up. Among ICD recipients, quinidine therapy was associated with a marked decline in appropriate and inappropriate shocks. Anguera et al. [15] reported a reduction from six shocks to no shocks per patient (*p* < 0.0001), and Mazzanti et al. [6] observed a fall in the incidence of patients with recurrent life-threatening arrhythmic events from 14.7% to 3.9% (*p* = 0.03). The randomised QUIDAM trial further reported no arrhythmic events during active hydroquinidine therapy compared with two events on placebo.

Although efficacy appears promising, tolerability remains the principal limitation to long-term use. Gastrointestinal symptoms, mainly diarrhoea and abdominal discomfort, occurred in 20–30% of patients and led to discontinuation in up to 25% [6,10,11,12]. QT-interval prolongation was uncommon and generally reversible with dose reduction; serious proarrhythmic events were rarely reported [12,16]. These findings emphasise the need for careful dose titration, patient counselling, and routine ECG monitoring.

Despite encouraging results, available data are limited by small cohort sizes, heterogeneous endpoints, and predominance of observational designs. Variability in dosing (200–738 mg/day), formulations (quinidine sulphate, bisulphate, hydroquinidine), and regional availability further complicates direct comparison. Consequently, multicentre prospective studies are needed to clarify optimal dosing regimens, long-term safety, and the comparative role of quinidine versus alternative non-device therapies such as catheter ablation.

From a clinical perspective, ICD therapy remains the gold standard for prevention of SCD in BrS patients at high risk (secondary prevention; selected high-risk primary prevention). Epicardial substrate ablation targeting arrhythmogenic right-ventricular outflow tract substrate can reduce ventricular arrhythmia burden and ICD shocks and may be preferred in patients with recurrent ventricular arrhythmias despite medical therapy or in those with frequent ICD shocks [23,24,25]. Quinidine and hydroquinidine remain valuable therapeutic options in four main contexts: (i) as alternatives to ICD implantation when contraindicated or declined, (ii) as adjuncts to ICD therapy to prevent recurrent shocks or electrical storm, (iii) as bridging therapy before ablation in unstable or refractory cases and (iv) in patients who are poor ablation candidates, decline ablation, or where ablation resources/expertise are unavailable [12,15,17]. These data support the continued consideration of quinidine or hydroquinidine in carefully selected BrS populations, provided that treatment is accompanied by ECG monitoring and careful assessment of tolerability.

## 5. Limitations

The findings of this narrative review should be interpreted in light of several limitations. First, only English-language studies were included, which may introduce selection bias. Second, most available evidence derives from small observational studies, registries, and case series rather than large randomized controlled trials, limiting the strength of the conclusions. Third, heterogeneity in dosing regimens, formulations, endpoints, and follow-up duration, limits direct comparability between studies. Fourth, inconsistent reporting of adverse events may have led to underestimation of true intolerance rates. Finally, differences in quinidine availability across countries may restrict the generalisability of these findings and influence treatment selection.

## 6. Conclusions

Quinidine-based therapy may reduce ventricular arrhythmic events and ICD interventions in selected patients with Brugada syndrome, particularly in secondary prevention of recurrent ICD shocks, or electrical storm. However, residual arrhythmic risk persists, and the current evidence base remains limited by small cohorts, heterogeneous endpoints, and predominantly observational data. Its use is also constrained by gastrointestinal intolerance and restricted drug availability. Future multicentre prospective studies are warranted to define optimal dosing, predictors of tolerance, and long-term clinical benefit.
